# Determination of Morpho-Physiological Traits for Assessing Drought Tolerance in Sugarcane

**DOI:** 10.3390/plants13081072

**Published:** 2024-04-11

**Authors:** Warodom Wirojsirasak, Patcharin Songsri, Nakorn Jongrungklang, Sithichoke Tangphatsornruang, Peeraya Klomsa-ard, Kittipat Ukoskit

**Affiliations:** 1 Department of Biotechnology, Faculty of Science and Technology, Rangsit Campus, Thammasat University, Pathum Thani 12120, Thailand; warodomw@mitrphol.com; 2 Mitr Phol Innovation and Research Center, Chaiyaphum 36110, Thailand; peerayak@mitrphol.com; 3 Department of Agronomy, Faculty of Agriculture, Khon Kaen University, Khon Kaen 40002, Thailand; nuntjo@kku.ac.th; 4 Northeast Thailand Cane and Sugar Research Center, Faculty of Agriculture, Khon Kaen University, Khon Kaen 40002, Thailand; 5 National Center for Genetic Engineering and Biotechnology, National Science and Technology Development Agency, Pathum Thani 12120, Thailand; sithichoke.tan@nstda.or.th

**Keywords:** sugarcane, drought tolerance, stay-green

## Abstract

Drought is a significant constraint to sugarcane productivity. Therefore, understanding how different varieties of sugarcane respond to drought stress can facilitate breeding programs and set up criteria for selecting drought-tolerant varieties. In the present study, we examined eight morpho-physiological traits to distinguish 40 sugarcane genotypes categorized into four groups based on significant differences in cane yield under non-stressed conditions and reduction of cane yield under drought-stressed conditions. The study was conducted during the formative stage in a greenhouse, encompassing both control and drought conditions. Drought treatments resulted in significant changes and differences in the mean values of various morpho-physiological traits. The hierarchical clustering analysis, utilizing stay-green traits such as higher chlorophyll fluorescence ratio (*F_v_*/*F_m_*), leaf chlorophyll content (SPAD), leaf relative water content (RWC), and lower leaf rolling score (LR), leaf drying score (LD), and drought recovery score (DR), successfully grouped 40 sugarcane genotypes into four major clusters, similar to the previously categorized groups. Correlation analysis showed significant relationships among cane yield, reduction of cane yield under drought conditions, and the stay-green traits. Our results demonstrated that morpho-physiological traits contributing to the “stay-green” phenotypes could be useful as selection criteria for drought tolerance in sugarcane.

## 1. Introduction

Sugarcane is an economically important crop cultivated globally, serving as a primary source of sucrose and bioenergy production [1]. However, sugarcane yield is frequently impacted by suboptimal growth conditions, particularly drought stress, during the formative growth stage, which is the most sensitive stage for moisture stress in sugarcane [2,3,4,5]. This is common in tropical regions [6], leading to substantial yield reductions by inhibiting growth and development, sometimes reaching up to 60% in rainfed areas [7]. Developing drought-tolerant sugarcane varieties with high yield potential and minimal reduction under drought stress is essential for enhancing the productivity and sustainability of rainfed sugarcane agriculture.

The development of cultivars with enhanced yield under drought stress has encountered relatively limited success for various reasons. Firstly, direct selection based on crop yield and yield reduction under drought conditions in comparison to well-watered conditions in a field environment is expensive, time-consuming, labor-intensive, and complex due to inherent genotype–environment interactions [8]. Secondly, the yield performance of plants in drought conditions is often masked by the spillover effects of high yield potential. Consequently, a high-yield variety often exhibits significant yields during drought, even though the relative yield reduction can be substantial [9]. A newly developed variety may not closely maintain its yield potential under drought stress. Employing molecular markers for the selection of drought-tolerant sugarcane genotypes is a promising approach to enhance breeding efficiency and precision. Various molecular markers, such as random amplified polymorphic DNA (RAPD; [10]), inter simple sequence repeat (ISSR; [11]), and amplified fragment length polymorphism (AFLP; [12]), have been used in identifying and selecting drought-tolerant sugarcane genotypes. However, the complex genetics of sugarcane, characterized by its polyploid and aneuploid genome, present significant challenges for molecular breeding efforts. Analytical approaches prioritizing breeding for high yield potential and minimal reduction through indirect selection strategies using morpho-physiological traits have garnered increasing attention in sugarcane breeding programs [9,13,14,15,16,17]. Ideally, these traits should exhibit strong correlations with tolerance to drought stress and yield-related traits. Additionally, they should be non-destructive, easily measurable in early phenological stages, and possess high heritability and repeatability to facilitate selection in breeding populations [9,18,19].

Although the mechanisms of drought tolerance in sugarcane are not entirely understood, certain morpho-physiological traits have been implicated in better performance under drought stress [7]. For instance, a higher chlorophyll fluorescence ratio (*F_v_*/*F_m_*), higher leaf chlorophyll content estimated via the SPAD index, higher leaf relative water content (RWC), higher transpiration rate, and lower leaf temperature can indicate a genotype with better performance [14,20]. Genotypes exhibiting earlier symptoms, such as leaf rolling (LR) and stomatal closure, are considered sensitive, while tolerant genotypes show the same symptoms after moderate stress [7,21]. Retaining green leaf area, known as the stay-green phenomenon, is crucial for maintaining yield [22,23]. Root characteristics also play a crucial role in evaluating the adaptive ability of sugarcane to drought stress [24,25]. Deeper root systems, characterized by traits such as rope roots and larger root systems, including root volume, total root length, and root biomass, are indicative of drought-tolerant genotypes. Deeper and more extensive root systems enable plants to access water and nutrients from deeper soil layers, thus enhancing their resilience to drought conditions [26,27]. Morpho-physiological traits that can classify tolerant and susceptible genotypes based on relative yield under drought conditions have been suggested as indirect selection indicators [28]. Identifying drought-tolerant varieties based on a single trait is challenging due to the complex genetic mechanisms underlying the plant’s response to drought stress [29]. Many studies conducted in this area have been limited by factors such as field conditions and a small number of genotypes, which may not fully capture the variability in drought response across different genetic backgrounds. Understanding how these traits change over time in response to water stress and recovery periods with a diverse range of genotypes is crucial for identifying reliable selection criteria for breeding drought-tolerant sugarcane varieties. 

This study aimed to identify morpho-physiological traits during the early growth stage to facilitate the rapid selection of drought-tolerant genotypes, focusing on achieving high yield and minimal reduction under drought stress. We investigated eight morpho-physiological traits, including *F_v_*/*F_m_* ratio, SPAD index, RWC, LR, leaf drying (LD), drought recovery index (DR), height, and shoot growth rate to differentiate sugarcane genotypes based on high yield potential and low reduction (P_H_R_L_) under drought conditions from other groups. These groups consist of those with high yield potential and high reduction (P_H_R_H_), low yield potential and high reduction (P_L_R_H_), and low yield potential and low reduction (P_L_R_L_). The study focused on drought and recovery responses during the formative growth phase in a greenhouse pot experiment. Hierarchical clustering was utilized to depict distinct drought-responsive patterns among sugarcane genotypes and to identify multiple traits associated with drought tolerance, particularly those linked to high yield potential and low reduction characteristics. Our findings highlight the utility of the morpho-physiological traits related to “stay-green” phenotypes as valuable selection criteria for drought tolerance in sugarcane.

## 2. Results

### 2.1. Morpho-Physiological Variations in Progressive Drought Stress and Recovery

**Figure 1:**
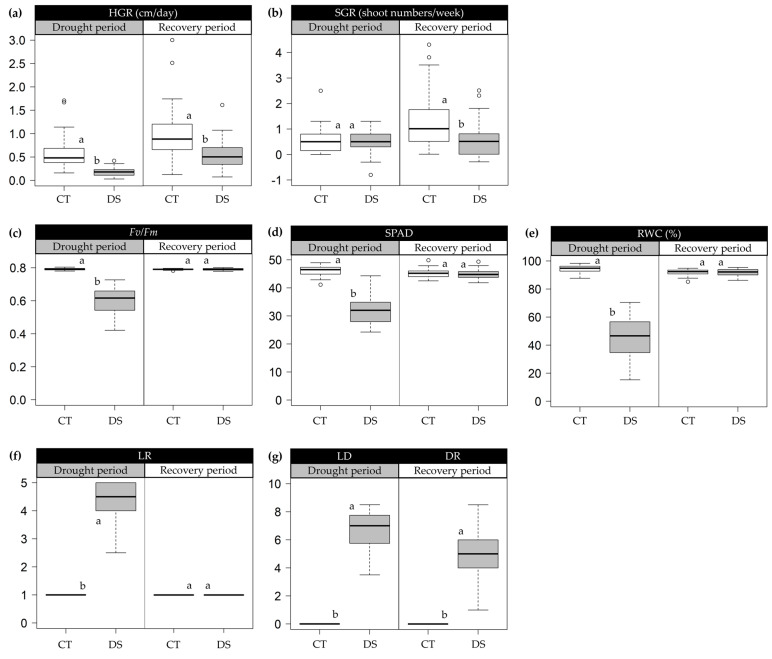
Box plots showing the descriptive statistics of morpho-physiological traits measured under control (CT) and drought (DS) conditions. (**a**) HGR (height growth rate); (**b**) SGR (shoot growth rate); (**c**) *F_v_*/*F_m_* (the chlorophyll fluorescence ratio); (**d**) SPAD (estimated chlorophyll content using SPAD units); (**e**) RWC (leaf relative water content); (**f**) LR (leaf rolling score); (**g**) LD (leaf drying score) and DR (drought recovery score). Different letters on the boxes indicate significant differences by the least significant difference (LSD) test at *p* < 0.05. The box’s horizontal line represents the median. The lower and upper limit of the box, lower and upper whisker, represents Q1 (25th percentile), Q3 (75th percentile), (Q1−1.5IQR), and (Q3 + 1.5IQR), respectively. IQR—interquartile range.

In this study, 40 sugarcane genotypes categorized into four groups, namely high yield potential and low reduction (P_H_R_L_), high yield potential and high reduction (P_H_R_H_), low yield potential and high reduction (P_L_R_H_), and low yield potential and low reduction (P_L_R_L_) (Supplementary Materials Figure S1), were grown in a greenhouse pot environment under both control (CT) and drought (DS) conditions. Under CT conditions, soil moisture content (SMC) ranged from 32.29 to 36.42% throughout the experiment. In contrast, under the DS condition, SMC dropped to 13.85% and 9.21% at 7 and 14 days after water withholding (DAW) during the drought period, respectively. Subsequently, SMC rebounded to 36.45% and 35.54% at 7 and 14 days after re-watering (DAR) during the recovery period, respectively (Supplementary Material Figure S2). Despite the short duration of the drought and recovery periods, significantly different and wide variations were observed among the studied genotypes for all traits (Figure 1 and Supplementary Material Figure S3).

During the drought period (91–104 days after planting; DAP), the intensity of drought-related symptoms in morpho-physiological traits was linked to decreased soil moisture content (Supplementary Material Figure S3). All traits significantly decreased under DS conditions, except for leaf rolling score (LR) and leaf drying score (LD), which significantly increased. However, shoot growth rate (SGR) showed a non-significant difference between the DS and CT conditions.

A higher reduction in the DS condition compared to the CT conditions was observed for height growth rate (HGR, 69.99%), leaf relative water content (RWC, 52.23%), estimated chlorophyll content (SPAD, 29.33%), chlorophyll fluorescence ratio (*F_v_*/*F_m_*, 24.97%), and SGR (9.88%), while LR (87.80%) and LD (74.78%) increased in the DS condition (Figure 1). In contrast, all traits showed a non-significant difference between the DS and CT conditions during the recovery period (105–118 DAP), except for HGR, SGR, and drought recovery score (DR) (Figure 1). A comparatively higher decrease was recorded in plant growth-related traits than in the other traits. HGR decreased by 44.96%, and SGR decreased by 48.73%, while *F_v_*/*F_m_*, SPAD, and RWC showed marginal variations of 0.02%, 0.32%, and 0.24%, respectively. However, DR related to the green leaf area of the plant decreased by 52.78%. 

**Figure 2:**
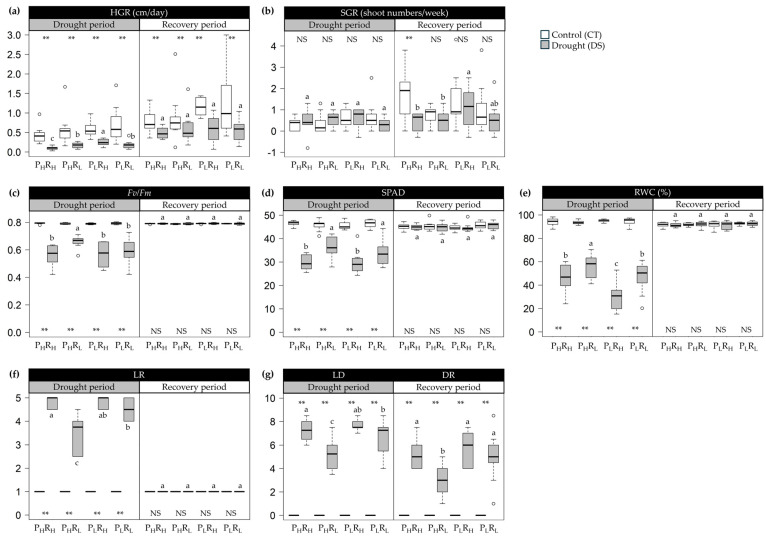
Box plots illustrating descriptive statistics in morpho-physiological traits under control (CT) and drought (DS) conditions of four sugarcane groups: high yield potential and high reduction (P_H_R_H_), high yield potential and low reduction (P_H_R_L_), low yield potential and high reduction (P_L_R_H_), and low yield potential and low reduction (P_L_R_L_). (**a**) HGR (height growth rate); (**b**) SGR (shoot growth rate); (**c**) *F_v_*/*F_m_* (the chlorophyll fluorescence ratio); (**d**) SPAD (estimated chlorophyll content using SPAD units); (**e**) RWC (leaf relative water content); (**f**) LR (leaf rolling score); (**g**) LD (leaf drying score) and DR (drought recovery score). ** and NS denote significant and non-significant variations between treatments at 0.01 probability level, respectively. Different letters on the boxes indicate significant differences by the least significant difference (LSD) test at *p* < 0.05. The box’s horizontal line represents the median. The lower and upper limit of the box, lower and upper whisker, represents Q1 (25th percentile), Q3 (75th percentile), (Q1−1.5IQR), and (Q3 + 1.5IQR), respectively. IQR—interquartile range.

Variations in morpho-physiological traits among sugarcane groups were observed during the drought and recovery periods (Figure 2). In the drought period, all traits exhibited significant differences among groups and between conditions, except for SGR. The P_L_R_H_ group showed the highest value in HGR at 0.25 cm/day, while the lowest value at 0.10 cm/day was recorded in the P_H_R_H_ group. The P_H_R_L_ group showed the highest values in *F_v_*/*F_m_* at 0.661, SPAD at 36.5, and RWC at 55.75%, but the lowest values in LR at 3.5 and LD at 5.2. In contrast, the P_L_R_H_ group displayed the lowest values in *F_v_*/*F_m_* at 0.562, SPAD at 29.62, and RWC at 30.45%, recording the highest values in LR at 4.9 and LD at 7.7. During the recovery period, all traits showed no significant differences among groups and conditions, except for the DR traits. The P_H_R_L_ group had the lowest DR value (3.0), whereas the P_L_R_H_ group recorded the highest value (5.8). HGR, *F_v_*/*F_m_*, SPAD, RWC, LR, and LD explained the differences among sugarcane groups and between conditions during drought, while DR explained the differences during recovery.

### 2.2. Hierarchical Clustering of Sugarcane Genotypes Based on Morpho-Physiological Traits

**Figure 3:**
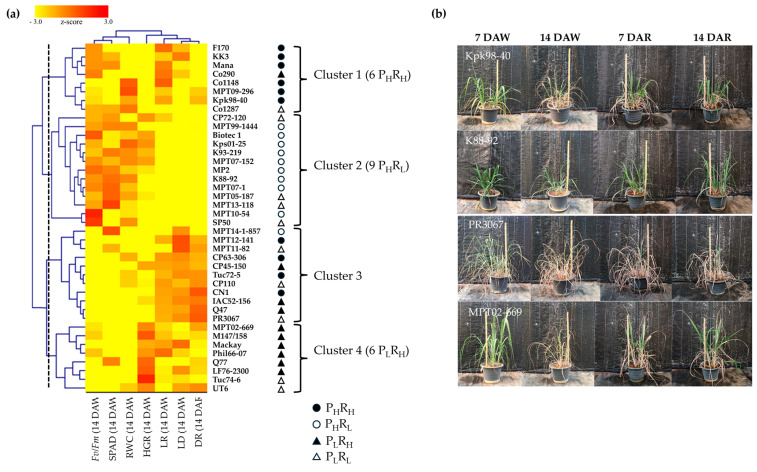
(**a**) Heatmap and clustering of 40 sugarcane genotypes based on seven morphological and physiological traits under drought conditions determined over 14 days of water withholding followed by 14 days of re-watering. (**b**) Phenotypic responses of the represented sugarcane variety for each cluster during the formative growth phase of sugarcane to drought stress at seven (7 DAW) and 14 (14 DAW) days of water withholding and water recovery at seven (7 DAR) and 14 (14 DAR) days of re−watering. Kpk98−40, K88−92, PR3067, and MPT02−669 represent clusters 1, 2, 3, and 4, respectively. HGR = height growth rate; SGR = shoot growth rate; *F_v_*/*F_m_* = the chlorophyll fluorescence ratio; SPAD = estimated chlorophyll content using SPAD units; RWC = leaf relative water content; LR = leaf rolling score; LD = leaf drying score; DR = drought recovery score; P_H_R_H_ = high yield potential and high reduction; P_H_R_L_ = high yield potential and low reduction; P_L_R_H_ = low yield potential and high reduction; P_L_R_L_ = low yield potential and low reduction.

Hierarchical clustering analysis using the UPGMA algorithm and a heatmap was conducted to cluster sugarcane genotypes based on their responses to drought and recovery treatments. This analysis utilized six traits (HGR, *F_v_*/*F_m_*, SPAD, RWC, LR, and LD) measured at 14 DAW and one trait (DR) measured at 14 DAR. As a result, 40 sugarcane genotypes were reclassified into four clusters, each reflecting distinct responses to drought conditions (Figure 3a). Genotypes with similar morpho-physiological responses to drought stresses were grouped together within the same cluster. Cluster 2 contained the highest number of sugarcane genotypes (13), followed by cluster 3 (11), 1 (8), and 4 (8). In general, cluster 1 was characterized by *F_v_*/*F_m_*, RWC, LR, and LD. Cluster 2 was dominated by *F_v_*/*F_m_*, SPAD, and RWC. LR, LD, and DR characterized cluster 3, while HGR, LR, and LD determined cluster 4. The first phenotype was represented by sugarcane genotypes in cluster 2, exhibiting a stay-green phenotype, sustaining their green leaf area throughout the experiment (Figure 3b). The second and third phenotypes were represented by clusters 1 and 4, respectively. Sugarcane genotypes in both clusters showed symptoms of leaf rolling and drying during the drought period but rapidly recovered after re-watering. However, genotypes in cluster 1 maintained *F_v_*/*F_m_* and RWC during the drought period, while HGR characterized cluster 4. The last phenotype was denoted by genotypes in cluster 3, where all morpho-physiological traits were severely affected by drought stress, and the green leaf area gradually recovered upon re-watering after withholding. Furthermore, the results revealed that the six genotypes in cluster 1 belonged to the P_H_R_H_ group, while the nine genotypes in cluster 2 matched the P_H_R_L_ group (Figure 3a). The six genotypes in cluster 4 were placed in the P_L_R_H_ group. However, the genotypes in cluster 3 included sugarcane genotypes from P_H_R_H_ (4), P_H_R_L_ (1), P_L_R_H_ (3), and P_L_R_L_ (3) groups.

### 2.3. Variability of the Genotypes in the Clusters

**Figure 4:**
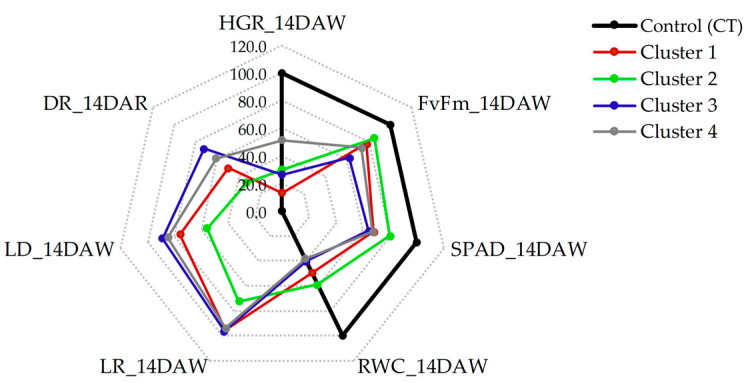
Radar plot showing changes in morphological and physiological traits of genotypes from different clusters due to drought treatments. Values are expressed as a percentage of trait values under DS conditions relative to those under CT conditions. HGR = height growth rate; *F_v_*/*F_m_* = the chlorophyll fluorescence ratio; SPAD = estimated chlorophyll content using SPAD units; RWC = leaf relative water content; LR = leaf rolling score; LD = leaf drying score; DR = drought recovery score.

The effects of drought treatment revealed that genotypes in cluster 2 exhibited significantly better performance under DS conditions, followed by those in clusters 1 and 4. In contrast, genotypes in cluster 3 were substantially affected by water stress (Figure 4 and Table 1). During the drought stress treatment, HGR decreased by 85.19%, 66.34%, 75.00%, and 56.52% in clusters 1, 2, 3, and 4, respectively, compared to CT conditions (Table 1). The percentage decrease in *F_v_*/*F_m_* was 21.84%, 15.15%, 38.08%, and 26.14% in clusters 1, 2, 3, and 4, respectively. A similar decrease pattern was observed in SPAD with 32.98%, 19.69%, 35.65%, and 32.83% decreases in clusters 1, 2, 3, and 4, respectively. As a result of drought stress treatment, the smallest decrease in RWC (41.02%) was recorded in cluster 2, followed by 49.51% (cluster 1), 59.96% (cluster 3), and 62.31% (cluster 4). Conversely, LR increased under drought stress treatment in all clusters. The lowest LR was recorded in cluster 2 (3.6), followed by cluster 3 (4.7), while the highest scores of LR (4.8) were recorded in the other two clusters. Meanwhile, the lowest LD was recorded in cluster 2 (5.0), followed by clusters 1 (6.8), 4 (7.6), and 3 (8.0). Under recovery treatment, the lowest DR was recorded in cluster 2 (3.0), followed by clusters 1 (4.5), 4 (5.5), and 3 (6.5).

Based on data from the field experiment, the potential yield under non-stressed experiments was recorded as the highest in cluster 2 (122.14 ton/ha.), followed by clusters 1 (121.20 ton/ha.), 3 (115.19 ton/ha), and 4 (100.98 ton/ha) (Table 1). Under the drought-stressed field experiment, the highest cane yield was observed in cluster 2 (106.84 ton/ha), followed by cluster 3 (67.27 ton/ha), 1 (65.27 ton/ha), and 4 (61.04 ton/ha). The percent reduction in cane yield was 12.53%, 39.55%, 41.60%, and 46.15% in clusters 2, 4, 3, and 1, respectively.

### 2.4. Principal Component Analysis

**Figure 5:**
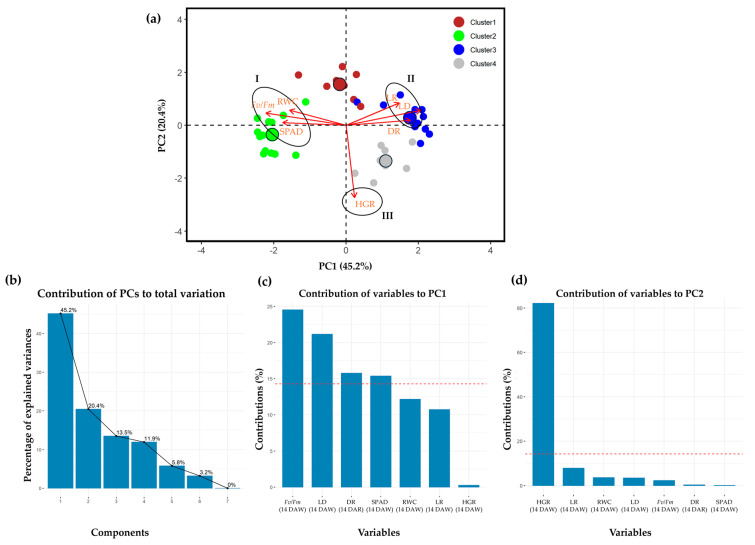
(**a**) Principal component analysis (PCA)−biplot of 40 sugarcane genotypes on the variation in seven morpho-physiological traits grown under drought conditions. Arrows indicate the strength of the trait influence on the first two PCs, with longer arrows representing a higher contribution of the traits. I, II, and III indicate the group of associated traits contributing to a cluster of sugarcane genotypes. Bigger circles indicate the centroid of the corresponding cluster. (**b**) Bar plots with percentage variation above represent the contribution of each PC to the total variation. (**c**,**d**) Red dashed lines in the bar plots denote reference lines, and the variable bars above the reference lines are considered the most important contributors to PC1 and PC2. HGR = height growth rate; *F_v_*/*F_m_* = the chlorophyll fluorescence ratio; SPAD = estimated chlorophyll content using SPAD units; RWC = leaf relative water content; LR = leaf rolling score; LD = leaf drying score; DR = drought recovery score.

Principle component analysis (PCA) was conducted on a dataset comprising 40 sugarcane genotypes, 6 traits (HGR, *F_v_*/*F_m_*, SPAD, RWC, LR, and LD) measured at 14 DAW, and 1 trait (DR) measured at 14 DAR to evaluate the diversity of the genotypes and their association with the observed traits. The PCA-biplot was generated, considering that the first two PCs accounted for the highest proportion of variance (Figure 5b). The first PC, explaining 45.2% of the total variability, was mainly contributed to by *F_v_*/*F_m_* (24.5%), LD (21.2%), DR (15.8%), and SPAD (15.4%) (Figure 5c). The second PC, explaining 20.4% of the total variation, was primarily defined by HGR (82.2%) (Figure 5d). The PCA-biplot analysis grouped traits based on homogeneity and dissimilarity. In this dataset, three groups of traits and four groups of genotypes were identified in the PCA biplot (Figure 5a). The *F_v_*/*F_m_*, SPAD, and RWC were clustered in group I, while LR, LD, and DR were in group II and HGR in group III. The PCA biplot revealed a strong association between group I traits and genotypes of cluster 2, group II traits with genotypes of cluster 3, and group III traits with genotypes of cluster 4. However, some traits of group I (RWC) and group II (LR) were closely linked with the genotypes of cluster 1.

### 2.5. Correlation Analysis

**Figure 6:**
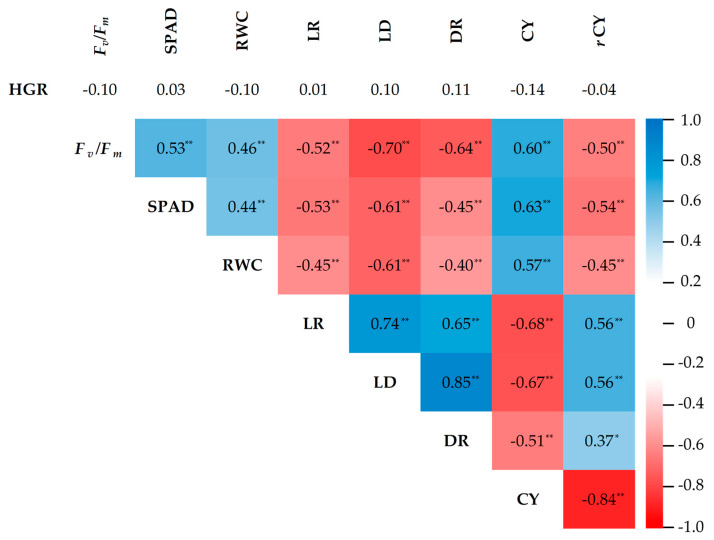
The correlation coefficient among morpho-physiological traits, cane yield, and the percentage reduction of cane yield was measured in 40 sugarcane genotypes under drought conditions. * and ** indicate significance at *p* < 0.05 and *p* < 0.01, respectively. HGR = height growth rate; *F_v_*/*F_m_* = the chlorophyll fluorescence ratio; SPAD = estimated chlorophyll content using SPAD units; RWC = leaf relative water content; LR = leaf rolling score; LD = leaf drying score; DR = drought recovery score; CY = cane yield under drought-stressed conditions and *r*CY = the percentage reduction of cane yield.

The Pearson correlation coefficient was determined to reveal the degree of relationships among seven morpho-physiological traits (HGR, *F_v_*/*F_m_*, SPAD, RWC, LR, LD, and DR). All traits showed significant correlations (*p* < 0.05) among them, except for the correlation between HGR and all morpho-physiological traits (Figure 6). Leaf drying (LD) exhibited the highest significant correlation with *F_v_*/*F_m_* (−0.70), SPAD (−0.61), RWC (−0.61), LR (0.74), and DR (0.85). Furthermore, the traits in group I (*F_v_*/*F_m_*, SPAD, and RWC) demonstrated a positive and significant correlation among them, while the traits in group II (LR, LD, and DR) showed a similar correlation pattern. Significantly negative correlations were noticed between the traits of groups I and II.

Cane yield (CY) exhibited positive and significant correlations (*p* < 0.05) with traits measured during the drought period, including *F_v_*/*F_m_* (0.60), SPAD (0.63), and RWC (0.57). Conversely, a negative and significant correlation was observed with LR (−0.68) and LD (−0.67) (Figure 6). The percentage reduction of cane yield (*r*CY) exhibited negative and significant correlations with *F_v_*/*F_m_* (−0.50), SPAD (−0.54), and RWC (−0.45) while showing positive and significant correlations with LR (0.56) and LD (0.57). However, CY and *r*CY showed a non-significant correlation with HGR. During the recovery period, DR exhibited significant correlations with CY (0.37) and *r*CY (−0.84). The results indicated that *Fv*/*Fm*, SPAD, RWC, LR, LD, and DR were not only associated with drought tolerance, represented by the sugarcane genotype in cluster 2 (Figure 3), but also with cane yield parameters.

## 3. Discussion

### 3.1. Trait Variability under Drought and Recovery Treatments

The present study evaluated the morpho-physiological traits of sugarcane genotypes to identify trait variability in responses to drought and recovery treatments during the formative growth phase. The significant changes and differences for all the evaluated traits were observed among sugarcane genotypes, providing wide genetic variability and an opportunity for drought-tolerant improvement in sugarcane (Figure 1 and Figure 2). The results indicated that growth-related traits (HGR and SGR), photosynthesis-related traits (*F_v_*/*F_m_* and SPAD), green leaf area-related traits (LR and LD), and RWC progressively declined with exposure to drought during the formative phase. However, *F_v_*/*F_m_*, SPAD, RWC, and LR could fully recover after drought relief within a short-term period, in contrast to the lack of recovery observed in HGR, SGR, and DR. Similar findings for the HGR have been reported by previous research [30,31]. It has also been positively correlated with stalk dry weight in sugarcane under early season drought [6]. This correlation might partially explain how drought can reduce yield in sugarcane.

The four sugarcane groups, namely P_H_R_H_, P_H_R_L_, P_L_R_H_, and P_L_R_L_, exhibited significant differences in both potential yield and percentage reduction in yield, reflecting variations in their levels of drought tolerance. The results indicated that sugarcane genotypes in the P_H_R_L_ group were less affected by drought than in other groups. They showed minimal reductions in photosynthesis-related traits and RWC and maintained a higher green leaf area throughout the experiment, attributed to lower LR, LD, and DR (Figure 2). In contrast, sugarcane genotypes belonging to the P_L_R_H_ group experienced severe effects under drought stress, with the green leaf area gradually recovering upon re-watering after withholding. Generally, genotypes that can delay drought symptoms, including leaf rolling, leaf senescence, and impairment of photosynthesis and growth, are regarded as more tolerant. Conversely, genotypes showing earlier symptoms are considered sensitive to drought [7,21]. The increase in photosynthetic attributes, leaf relative water content, and green leaf area in drought-tolerant genotypes relative to drought-sensitive genotypes has been observed in sugarcane [14,32,33], switchgrass [34,35], wheat [36,37], and rice [38,39]. Tolerance mechanisms are favorable under mild and moderate drought conditions because they allow plants to endure stress, maintain essential functions, and sustain growth during stress [40,41]. The present study demonstrated that higher values of *F_v_*/*F_m_*, SPAD, and RWC, coupled with lower values of LR, LD, and DR, reflected the higher capability to tolerate drought stress. HGR, *F_v_*/*F_m_*, SPAD, RWC, LR, and LD played an important role in screening drought-tolerant genotypes during the drought period. Meanwhile, during the recovery phase, monitoring DR becomes essential as it reflects the plants’ ability to recover from stress once water is reintroduced. These traits exhibited varied responses to stress across different sugarcane groups and conditions, allowing for the distinction between sugarcane genotypes known to be drought-tolerant or susceptible.

### 3.2. Relationships between Genotypes, Traits, and Drought Tolerance

Drought simultaneously affects various morphological and physiological traits in plants. A single characteristic cannot reflect the complexity of drought tolerance mechanisms, so it is necessary to consider multiple traits together to select a drought-tolerant genotype. Based on the proposed morpho-physiological traits, hierarchical clustering analysis can provide insights into the interrelationships between genotypes and measured traits under various treatments [35,42]. As the result of cluster analysis, the 40 sugarcane genotypes were regrouped into four distinct hierarchical clusters (clusters 1–4), each exhibiting different responses to drought conditions. The heat map and radar plot revealed that genotypes in cluster 2 demonstrated the highest ability to tolerate water stress, as indicated by all evaluated traits, except HGR (Figure 3 and Figure 4 and Table 1). These genotypes can minimize reductions in *F_v_*/*F_m_*, SPAD, RWC, LR, LD, and DR, allowing them to maintain their yield potential. The results of PCA also revealed significant contributions from these traits in characterizing variations in the sugarcane genotypes. *F_v_*/*F_m_*, SPAD, and RWC clustered in the PCA-biplot, closely scattered around sugarcane genotypes in cluster 2. Conversely, traits LR, LD, and DR formed a cluster and were closely associated with sugarcane genotypes in cluster 3, while HGR contributed substantially to the differentiation in cluster 4. These results underscore the importance of these traits in selecting characteristics under drought conditions, supporting the identification of drought-tolerant genotypes of cluster 2.

In the present study, Pearson correlation analysis revealed significant correlations among cane yield under drought conditions, the percentage reduction of cane yield, and morpho-physiological traits, such as *F_v_*/*F_m_*, SPAD, RWC, LR, LD, and DR. These correlations underscore the substantial role of these traits in fostering high yield under drought conditions and contribute to the clustering of sugarcane genotypes into similar groups as those pre-categorized. Consistent with our findings, results from various studies have also reported that high values of *F_v_*/*F_m_*, SPAD, and RWC, along with low leaf rolling and drying scores, were related to better yield or yield stability under drought stress [43,44,45,46,47,48]. Traits linked to drought tolerance, which exhibit direct correlations with cane yield, are typically employed to identify superior genotypes or cultivars [49,50,51]. Under drought conditions, traits in group I (*F_v_*/*F_m_*, SPAD, and RWC) showed significant positive correlations with each other but exhibited negative correlations with traits in group II (LR, LD, and DR). This implies that genotypes maintaining their water status may mitigate damage to the chloroplast by improving antioxidant enzyme activity. This process could delay drought-induced leaf senescence, enhancing photosynthetic capability under drought conditions [24,42,52]. Photosynthesis in monocot leaves is affected directly by leaf structure. From light interception to carbon dioxide metabolic fixation, leaf rolling plays an essential role in regulating gas exchange and photosynthesis [53]. Leaf rolling is a plant mechanism to avoid excessive sunlight exposure; low water status causes the turgor pressure to drop and the bulliform cells to deflate, reducing stomatal conductance, photosynthesis, and yield [54]. Bulliform cells enlarge to maintain turgor pressure, which flattens the leaves when the drought is relieved [55]. These findings thus reveal the prominence of these traits in selecting tolerant genotypes for drought stress.

### 3.3. Morpho-Physiological Traits Related to Stay-Green Phenotype

Drought can accelerate plant leaf senescence, resulting in decreases in biomass, green leaves, canopy size, and loss of photosynthesis due to diminished chlorophyll levels, ultimately leading to lower yields [56]. Genotypes with higher photosynthesis-related traits, RWC, and green leaf area-related traits can minimize the reduction in morpho-physiological and yield traits under drought conditions. The retention of green leaf area, often referred to as the “stay-green” phenomenon, has been reported as a crucial characteristic for sustaining yield [22,23]. A higher chlorophyll fluorescence ratio (*F_v_*/*F_m_*), elevated leaf chlorophyll content estimated via the SPAD index, increased leaf relative water content (RWC), a higher transpiration rate, and a lower leaf temperature can indicate a genotype with superior performance [14,20]. Leaf senescence correlates with leaf relative water content [57,58]. The mean relative water content of leaves in the non-senescent cultivar was higher than that of the senescent cultivar. Delayed leaf senescence in the stay-green phenotype provides substantial tolerance to plants, especially those exposed to drought stress [59], by reprogramming the expression of genes controlling steps towards chlorophyll catabolism and leaf death [60]. It is considered an important trait that allows plants to retain their leaves in an active photosynthetic state when subjected to stress conditions. Upon receiving stress signals, late-senescent plants can adapt to maintaining high water potential in tissues to avoid dehydration stress and allow normal cell functionality. In sugarcane, drought-tolerant genotypes display higher values for SPAD, *F_v_*/*F_m_*, and RWC than drought-susceptible genotypes [14], supporting the conclusion that leaf drying (LD) exhibited the highest significant correlation with SPAD, *F_v_*/*F_m_*, RWC, and DR. Our study provided information about morpho-physiological traits which play an important role in imparting stay-green trait, which has potential to stabilize the yield of sugarcane under drought stress condition. These morpho-physiological traits are non-destructive, rapid, and easily measurable in the early growth stage, inexpensive, and exhibit high repeatability, facilitating their use in the selection of breeding populations.

## 4. Materials and Methods

### 4.1. Plant Materials and Stress Treatment

Based on our previous research, we investigated the effects of drought stress on the yield components of 159 sugarcane genotypes, examining both non-stressed (NS) and drought-stressed (DS) treatments in the field experiments [61]. These genotypes exhibited varying degrees of drought tolerance, as evidenced by differences in cane yield and reductions in cane yield under drought conditions. In this study, we selected ten sugarcane genotypes representing each of the following groups: high yield potential and low reduction (P_H_R_L_), high yield potential and high reduction (P_H_R_H_), low yield potential and high reduction (P_L_R_H_), and low yield potential and low reduction (P_L_R_L_). These groups were categorized based on significant differences in cane yield under non-stressed conditions and reduction of cane yield under drought-stressed conditions, as illustrated in Figure S1. Seedlings of all genotypes were derived from single-budded setts and pre-germinated in plastic trays. The uniformly germinated seedlings 30 days after planting (DAP) were then transplanted into 15-inch plastic pots containing 15 kg of dry soil. The soil type was identified as sandy clay loam with a composition of 52.93% sand, 19.00% silt, and 28.07% clay. The soil water holding capacity (WHC) was determined to be 38.89%. The experiment was set up as a factorial design in a randomized complete block experiment, with three replications, under greenhouse conditions at the Mitr Phol Innovation and Research Center, Thailand, during the dry season (January to April 2019). Factor A included two water regimes (full irrigation and drought stress followed by recovery), and factor B comprised 40 sugarcane genotypes. Water was supplied daily at close WHC level from planting to 90 DAP. Subsequently, the water level at WHC was maintained throughout the experiment for control (CT) conditions. For drought (DS) conditions water was withheld during 91 to 104 DAP and then re-watering during 105–118 DAP at the WHC level.

### 4.2. Soil Moisture Measurement and Meteorological Conditions

Soil moisture content (SMC) was measured weekly between 91 and 118 DAP using the gravimetric method at the midpoint of soil depths (Supplementary Materials Figure S2a). The soil samples were weighed and subjected to oven-drying at 105 °C for 48 h. The percentage of soil moisture was calculated by comparing the weights of the wet and dry soils using the following equation:

$$\mathrm{SMC}\;\left(\%\right)=\left(\frac{\mathrm{wet}\;\mathrm{soil}-\mathrm{dry}\;\mathrm{soil}}{\mathrm{dry}\;\mathrm{soil}}\right)\times 100.$$

Meteorological data, including relative humidity, maximum temperature, and minimum temperature, were collected daily between 91 and 118 DAP from the weather station in the greenhouse. Throughout the experiment, daily relative humidity ranged from 41.1% to 62.5%. The maximum daily air temperature ranged from 36.6 °C to 48.1 °C, while the minimum daily air temperature ranged from 21.6 °C to 26.2 °C (Supplementary Materials Figure S2b).

### 4.3. Morpho-Physiological Measurements

Data on morpho-physiological traits were collected five times at 7-day intervals for both control (CT) and drought (DS) treatments. Data collection covered intervals of 0, 7, and 14 days after withholding water (DAW) between 90 to 104 DAP and 7 and 14 days after re-watering (DAR) between 105 to 118 DAP. Growth-related traits were assessed through the growth rates of plant height and shoot numbers, referred to as height growth rate (HGR) and stem growth rate (SGR), respectively. Photosynthesis-related traits were measured through the photochemical efficiency of photosystem II, indicated by the chlorophyll fluorescence ratio (*F_v_*/*F_m_*), and estimated chlorophyll content using SPAD units (SPAD index). Green leaf area-related traits were assessed using leaf rolling score (LR), leaf drying score (LD), and drought recovery score (DR), while plant water status was evaluated through leaf relative water content (RWC).

Plant height was measured from the ground to the top visible dewlap (TVD) of the main stem, while the number of stems per pot was recorded by counting the total number of stems that emerged from the soil. The HGR and SGR were calculated as follows [6]:

$$\mathrm{HGR}=\left(\frac{\Delta H}{\Delta T}\right),$$

$$\mathrm{SGR}=\left(\frac{\Delta \mathrm{SN}}{\Delta T}\right),$$

where ∆H represents the difference in plant height between the two measurements (plant height at 104 DAP–height at 90 DAP), ∆T represents the time interval between the two measurements (104 DAP–90 DAP), and ∆SN represents the difference in stem numbers between the two measurements (stem numbers at 104 DAP–stem numbers at 90 DAP)

The *F_v_*/*F_m_* ratio was measured between approximately 09:00 a.m. and 2.00 p.m. on cloudless days using a chlorophyll fluorescence meter (Handy PEA, Hansatech Instrument Ltd., Norfolk, UK). The center of the leaf blade at the first and second fully expanded leaf of the main stem per plot was dark-adapted for 30 min using a leaf clip before fluorescence measurements. The *F_v_*/*F_m_* ratio was determined to quantify the level of drought-induced photoinhibition [62]. The variable fluorescence (*F_v_*) represents the difference between *F_o_* (the minimum fluorescence) and *F_m_* (the maximum fluorescence). Leaf chlorophyll content (SPAD unit) was estimated non-destructively using a SPAD chlorophyll meter (SPAD-501, Minolta, Tokyo, Japan). Measurements were taken on the same leaf with the *F_v_*/*F_m_* ratio between approximately 09:00 a.m. and 2.00 p.m. The average measurements at the bottom, middle, and tip of each leaf were recorded.

The relative water content (RWC) of plant leaves was examined [63]. Leaf samples, approximately 7 cm^2^ in size (1.0 cm × 7.0 cm), were cut and immediately placed in pre-weighted tubes kept in an icebox to minimize water loss. The fresh weight (FW) was then recorded, and the samples were immersed in distilled water for 24 h at 4 °C in darkness. After blotting the leaves dry, they were reweighed to obtain the turgid weight (TW). The dry weight (DW) was determined after drying in an oven at 80 °C for 48 h. The RWC was calculated as follows:

$$\mathrm{RWC}\;\left(\%\right)=\left[\frac{\left(\mathrm{FW}-\mathrm{DW}\right)}{\left(\mathrm{TW}-\mathrm{DW}\right)}\right]\times 100.$$

Leaf rolling (LR) in drought-stressed plants was characterized using a score scale from 1 (healthy or unrolled leaf) to 5 (onion leaf or tight rolling) [38]. Leaf drying (LD) was evaluated based on the dried leaf area, using a scoring system from 0 (no symptoms) to 9 (dead plant) [64]. The drought recovery score (DR) was utilized to indicate the percentage of leaf area that recovered to green after re-watering, employing a scoring system from 1 (90–100% recovery) to 9 (0–19% recovery) [39]. All evaluations were performed at noon using whole plants.

### 4.4. Statistical Analysis

The experimental data were subjected to an analysis of variance (ANOVA). Significant differences between means were determined based on the least significant difference (LSD) test at 0.05 probability level using Statistix 10 software program (Analytical Software, Tallahassee, FL, USA). R statistical software version 4.2.2 was employed to determine the Pearson correlation coefficient between traits and perform principal component analysis (PCA) [65]. In addition, hierarchical clustering and heatmap were conducted using the UPGMA algorithm in GENESIS software version 1.8.1 [66]. A radar plot was generated based on trait values under DS conditions, expressed as a percentage relative to those under CT conditions.

## Figures and Tables

**Table 1:** Mean of morphological and physiological traits and percent change over CT conditions for 40 sugarcane genotypes grown under both CT and DS conditions.

Cluster	Treatment	Drought Period						Recovery Period	Yield (ton/ha.)
		HGR	* F_v_* /*F_m_*	SPAD	RWC	LR	LD	DR	
1	CT	0.54	0.792	46.7	92.91	1.0	0	0	121.20
	DS	0.08 ^c^	0.619 ^b^	31.3 ^b^	46.91 ^ab^	4.8 ^a^	6.8 ^b^	4.5 ^b^	65.27 ^b^
	% Change	(-) 85.19 ^C^	(-) 21.84 ^B^	(-) 32.98 ^B^	(-) 49.51 ^AB^	96.0 ^A^	75.56 ^B^	50.0 ^B^	(-) 46.15 ^B^
2	CT	0.53	0.792	45.7	94.31	1.0	0	0	122.14
	DS	0.18 ^b^	0.672 ^a^	36.7 ^a^	55.62 ^a^	3.6 ^b^	5.0 ^c^	3.0 ^c^	106.84 ^a^
	% Change	(-) 66.34 ^AB^	(-) 15.15 ^A^	(-) 19.69 ^A^	(-) 41.02 ^A^	72.0 ^B^	55.56 ^C^	33.33 ^C^	(-) 12.53 ^A^
3	CT	0.60	0.793	46.0	94.31	1.0	0	0	115.19
	DS	0.15 ^b^	0.491 ^c^	29.6 ^b^	37.76 ^b^	4.8 ^a^	8.0 ^a^	6.5 ^a^	67.27 ^b^
	% Change	(-) 75.0 ^BC^	(-) 38.08 ^C^	(-) 35.65 ^B^	(-) 59.96 ^B^	96.0 ^A^	88.89 ^A^	72.22 ^A^	(-) 41.60 ^B^
4	CT	0.69	0.788	46.0	95.68	1.0	0	0	100.98
	DS	0.30 ^a^	0.582 ^b^	30.9 ^b^	36.06 ^b^	4.7 ^a^	7.6 ^a^	5.5 ^ab^	61.04 ^b^
	% Change	(-) 56.52 ^A^	(-) 26.14 ^B^	(-) 32.83 ^B^	(-) 62.31 ^B^	94.0 ^A^	84.44 ^A^	61.11 ^AB^	(-) 39.55 ^B^

Different letters indicate a significant difference by the least significant difference (LSD) test at *p* < 0.05. HGR = height growth rate; *F_v_*/*F_m_* = the chlorophyll fluorescence ratio; SPAD = estimated chlorophyll content using SPAD units; RWC = leaf relative water content; LR = leaf rolling score; LD = leaf drying score; DR = drought recovery score.

## Data Availability

Data are contained within the article or Supplementary Material.

## Supplementary Materials

The following supporting information can be downloaded at https://www.mdpi.com/article/10.3390/plants13081072/s1. Figure S1: The difference in cane yield and the percentage reduction in yield among 40 sugarcane genotypes under both non-stressed and drought-stressed conditions in field environments; Figure S2: (a) Soil moisture content (%) at the midpoint of soil depths, (b) maximum temperature (Tmax, °C), minimum temperature (Tmin, °C), and relative humidity (RH, %) during 91–118 days after planting under greenhouse environments; Figure S3: Dot plots showing the variation in eight measured traits of 40 sugarcane genotypes grown during drought and recovery periods under drought conditions.
