# Supplementary material for: Determination of Morpho-Physiological Traits for Assessing Drought Tolerance in Sugarcane
Source: Plants (Basel). 2024 Apr 11;13(8):1072. doi: 10.3390/plants13081072 (PMC11054708; doi:10.3390/plants13081072)

Figure S1. The difference in cane yield and the percentage reduction in yield among 40 sugarcane genotypes under both non-stressed and drought-stressed conditions in field environments [54]. Bars with different letters, as well as A and B, indicate a significant difference determined by the least significant difference (LSD) test at $p < 0.05$.

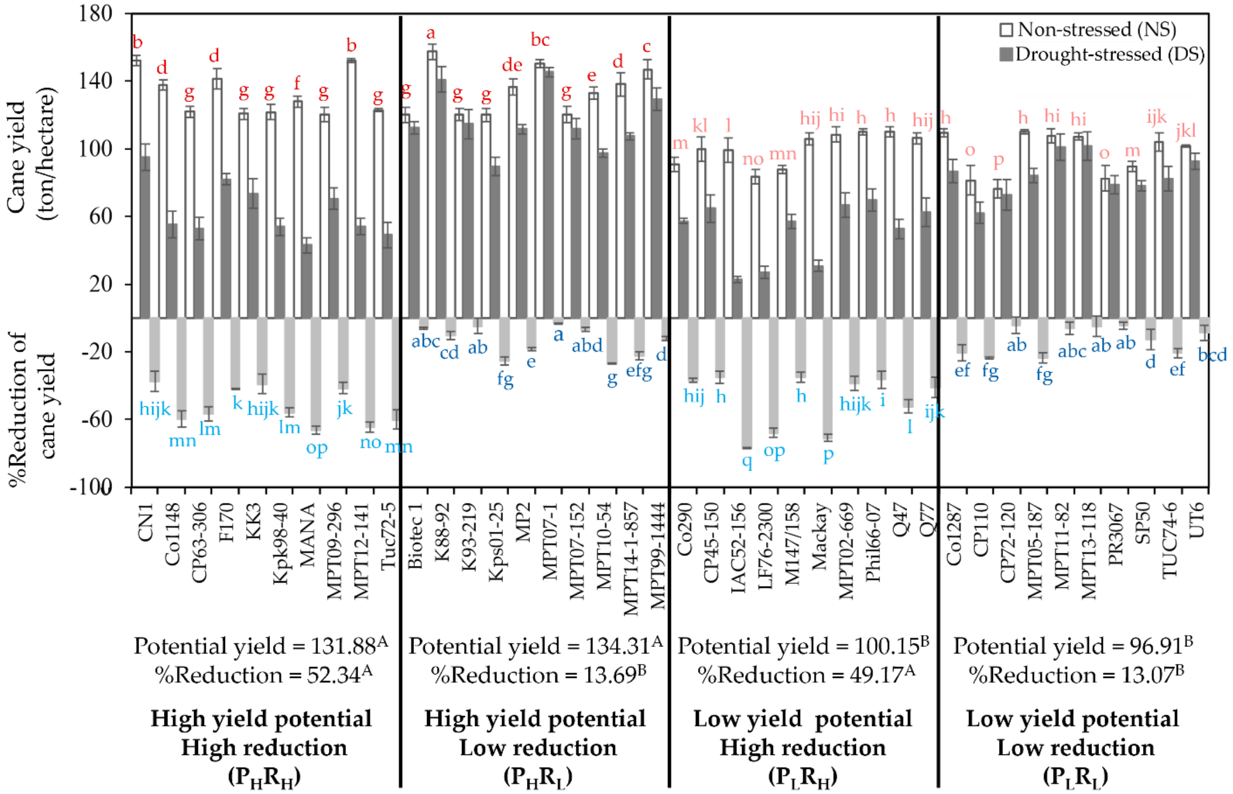

Figure S2. **(a)** Soil moisture content (%) at the midpoint of soil depths, **(b)** maximum temperature (Tmax, °C), minimum temperature (Tmin, °C), and relative humidity (RH, %) during 91-118 days after planting under greenhouse environments. Asterisks (*) indicate significant variations between treatments at the 0.05 probability level, while "NS" denotes non-significant variations.

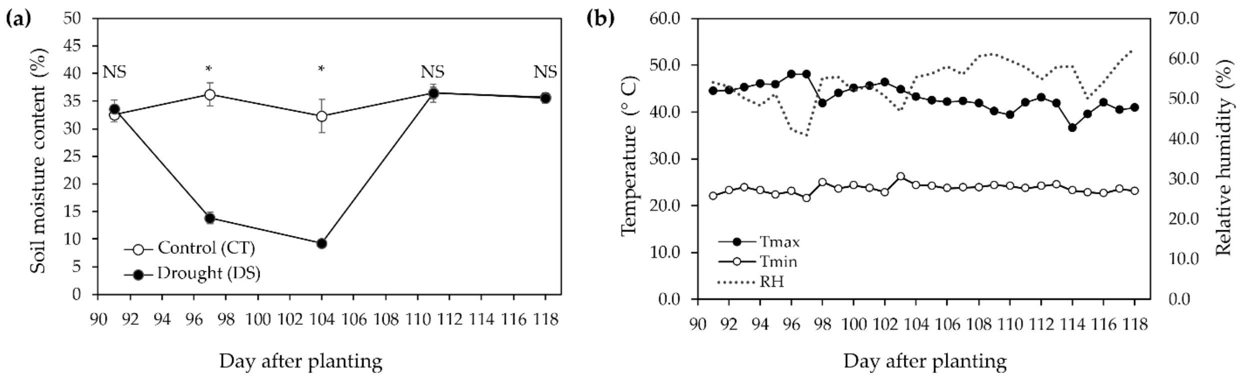

Figure S3. The black circle, white circle, black triangle, and white triangle on the boxes indicate the distribution of sugarcane genotypes in the groups of high yield potential and high reduction (P_HR_H), high yield potential and low reduction (P_HR_L), low yield potential and high reduction (P_LR_H), and low yield potential and low reduction (P_LR_L), respectively. HGR = height growth rate; SGR = shoot growth rate; F_v/F_m = the chlorophyll fluorescence ratio; SPAD = estimated chlorophyll content using SPAD units; RWC = leaf relative water content, LR = leaf rolling score; LD = leaf drying score; DR = drought recovery score

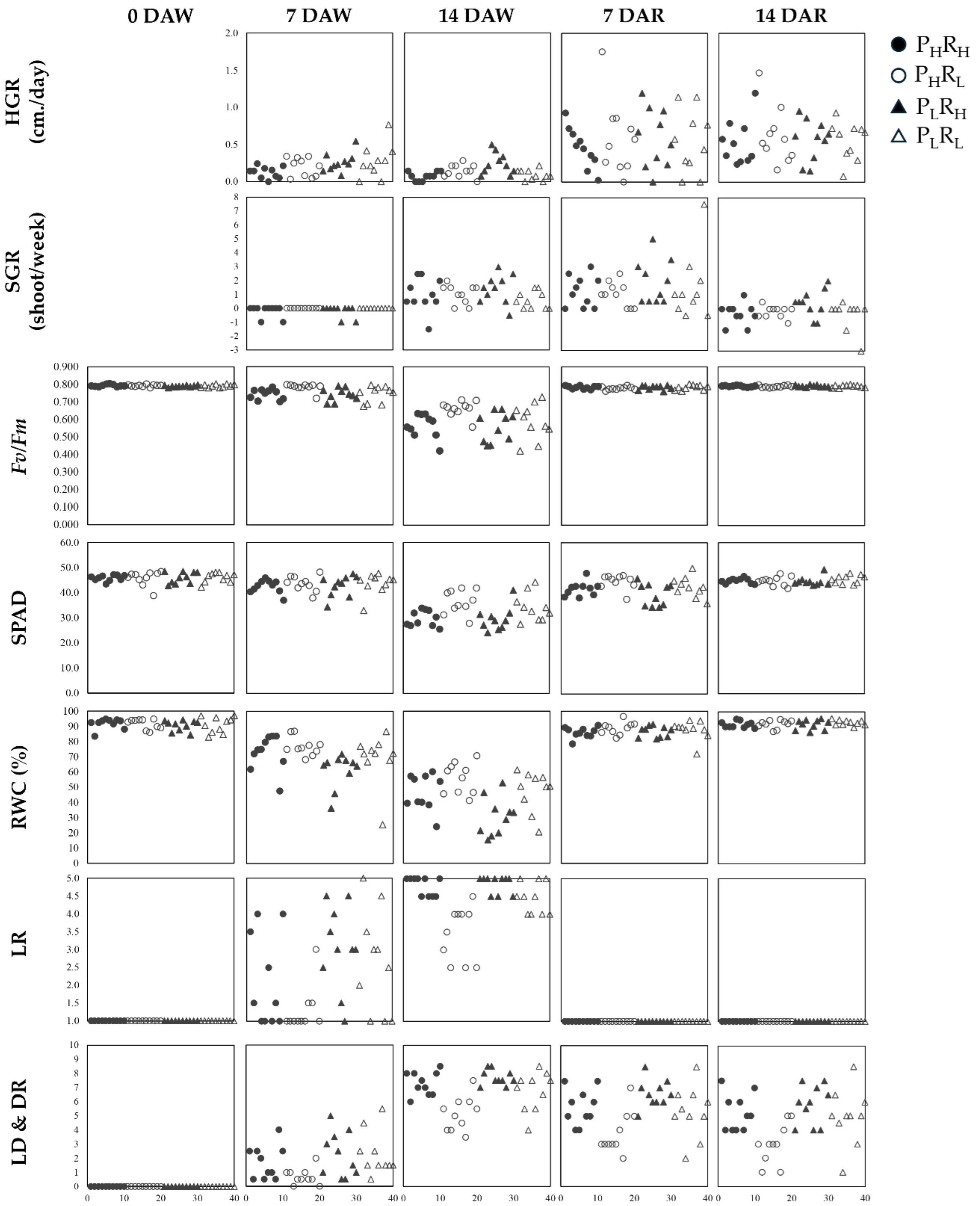

Supplement: Supplementary file 1 [file plants-13-01072-s001.zip › plants-2955054-supplementary.pdf]
